# Patterns of Obesity and Lymph Fluid Level during the First Year of Breast Cancer Treatment: A Prospective Study

**DOI:** 10.3390/jpm5030326

**Published:** 2015-09-03

**Authors:** Mei R. Fu, Deborah Axelrod, Amber A. Guth, Jason Fletcher, Jeanna M. Qiu, Joan Scagliola, Robin Kleinman, Caitlin E. Ryan, Nicholas Chan, Judith Haber

**Affiliations:** 1 College of Nursing, New York University, 433 First Avenue, New York, NY 10010, USA; E-Mails: jason.fletcher@nyu.edu (J.F.); qiujeanna@gmail.com (J.M.Q.); cer365@nyu.edu (C.E.R.); chilok91@gmail.com (N.C.); jh33@nyu.edu (J.H.); 2 Department of Surgery, School of Medicine, New York University, 160 East 34 Street, New York, NY 10016, USA; E-Mails: Deborah.Axelrod@nyumc.org (D.A.); Amber.Guth@nyumc.org (A.A.G.); 3 NYU Laura and Isaac Perlmutter Cancer Center, 160 East 34 Street, New York, NY 10016, USA; E-Mails: joan.scagliola@nyumc.org (J.S.); Robin.Kleinman@nyumc.org (R.K.)

**Keywords:** body mass index, breast cancer, lymphedema, lymph fluid, obesity, overweight

## Abstract

Obesity is one of the risk factors for developing lymphedema following breast cancer treatment. We prospectively enrolled 140 women and followed the participants for 12 months after surgery to investigate patterns of obesity and lymph fluid level in the first year of cancer treatment. Electrical bioimpedance devices were used to measure weight, BMI, and percent of body fat as well as lymph fluid level. General instructions were given to the participants on maintaining pre-surgery weight. Among the 140 participants, 136 completed the study with 2.9% attrition. More than 60% of the participants were obese (30.8%) or overweight (32.4%), while only two participants were underweight and about 35% had normal weight. This pattern of obesity and overweight was consistent at 4–8 weeks and 12 months post-surgery. At 12 months post-surgery, the majority of the women (72.1%) maintained pre-surgery weight and 15.4% had >5% weight loss; 12.5% of the women increase >5% of their weight. Significantly more patients in the obesity group had lymphedema defined by L-Dex ratio >7.1 than those in the normal/underweight and overweight group at pre-surgery and 4–8 weeks post-surgery. There was a trend of more patients in the obesity group had L-Dex ratio >7.1 at 12 months post-surgery. Obesity and overweight remain among women at the time of cancer diagnosis and the patterns of obesity and overweight continue during the first year of treatment. General instructions on having nutrition-balanced and portion-appropriate diet and physical activities daily or weekly can be effective to maintain pre-surgery weight.

## 1. Introduction

Worldwide obesity and breast cancer prevalence continues increasing [1,2].Obesity, as defined by body mass index [kg/m^2^] (BMI), is one of the established risk factors for breast cancer occurrence and recurrence [1,3]. Obesity is also negatively associated with disease-free survival and mortality from breast cancer [4,5].

Each year, about 1.38 million women are diagnosed with breast cancer worldwide [2]. Advances in cancer treatment have made it possible that over 90% of women treated for breast cancer have achieved five-year survival and currently more than 2.9 million breast cancer survivors are living in the United States [6]. With the increased rate and length of survival from breast cancer, more and more survivors are facing life-time risk of developing late effects of cancer treatment that negatively impact long-term survivorship [7].

Breast cancer-related lymphedema (hereafter, lymphedema) is one of the most distressing and feared late effects that usually occurs 1 to 5 years or even 20 years after cancer treatment [8,9,10]. Lymphedema can be a progressive and chronic accumulation of lymph fluid in the interstitial spaces of the affected limb and areas, leading to abnormal swelling [8,11]. Consequently, lymphedema remains a major health problem affecting many breast cancer survivors and exerting tremendous negative impact on survivors’ quality of life [7]. Although at present no surgery or medication can cure lymphedema, this condition can be managed with early and appropriate treatment.

The most commonly recognized non-modifiable risk factor for lymphedema are factors associated with breast cancer treatment, such as axillary lymph node dissection [8,9,10,11,12], mastectomy [13,14], extent of axillary surgery [6,13,14,15,16], number of positive lymph nodes [8,9,10,11,12,13], chemotherapy [13,14], and radiation [13,15]. A modifiable personal factor, such as obesity or BMI > 30 kg/m^2^, is identified as an important risk factor for lymphedema, however, research findings are conflicting [10,11,12,13,14,15,16,17]. For example, some studies suggest that only obesity defined by BMI ≥ 30 kg/m^2^ is associated with an increased risk of lymphedema, yet others report that being overweight as defined by BMI > 25– < 30 kg/m^2^ may also increase lymphedema risk [12]. Such conflicting findings in part are due to current study limitations, including retrospective assessment of lymphedema, small sample size, lack of pre-surgery limb volume or lymph fluid assessment, self-reported lymphedema, and self-reported weight or BMI [15,16,17,18,19,20,21].

The ongoing challenge to accurately detect and measure lymphedema is perhaps the most significant factor that contributes to conflicting findings in the literature due to different methods of defining, measuring, and quantifying lymphedema used in research [15,16,17,18,19,20,21]. Indirect methods measuring limb size, limb girth, or limb volume are usually used in the existing studies, including water displacement [20], infrared perometry [17,18,21], and sequential circumference limb tape measurement [22]. Such indirect methods do not discriminate bones, muscle, fat, or other soft tissues from lymph or extracellular fluid [23]. Misinterpretation or a false positive or negative lymphedema diagnosis may occur because of changes in muscle, fat, and soft tissues that cause inaccurate limb size or limb volume and circumferential limb measurements [23]. Furthermore, the criteria vary in determining the presence of lymphedema based on limb size or limb girth or limb volume. The most commonly accepted criterion of lymphedema is a 2-cm increase in limb girth or a 2-cm inter-limb difference, a 200 mL increase in limb volume or a 10% limb volume change [12,13,14,15,16,17,18,19,20,21,22,23]. Despite the known link between BMI and lymphedema measured by indirect measures of limb girth or limb size or limb volume, limited studies have explored the relationship between BMI and lymph fluid level as measured by bioelectrical impedance analysis (BIA). BIA provides an impedance ratio by assessing lymph fluid level through evaluating the impedance or opposition to the low frequency current measured in the unaffected limb divided by that of the affected or at risk limb, then is calculated as a Lymphedema Index termed as L-Dex ratio [23].

Lymphedema is the result of an accumulation of excess lymph fluid in the affected limb and obesity is an important risk factor for lymphedema [8,9,10,11], thus the purpose of the study was to prospectively investigate patterns of obesity and lymph fluid level in the first year of cancer treatment at the time points of pre-surgery, 4–8 weeks and 12 months post-surgery. The aims of the study were to explore: (1) patterns of obesity defined by BMI > 30 kg/m^2^; (2) patterns of lymph fluid level by BIA in terms of lymphedema index (L-Dex) ratio; (3) the relationship between obesity and lymph fluid level.

## 2. Methods

### 2.1. Ethical Consideration

This study was approved by the Institutional Review Board of NYU Langone Medical Center.

### 2.2. Study Design and Population

This was a prospective and longitudinal study with repeated-measures. The study population was women who were older than age 21, had a first time diagnosis of breast cancer (Stage I–III), and were scheduled for surgical treatment, including lumpectomy or mastectomy, sentinel lymph node biopsy or axillary lymph node dissection.

Exclusion criteria were women who were diagnosed with metastatic cancer (Stage IV), had a prior history of breast cancer and lymphedema, bilateral breast cancer, as well as women who had an artificial knee or hip, and kidney or heart failure, as the manufacturer suggests BIA may not be accurate under these conditions.

Between December 2011 and April 2014, we prospectively enrolled 140 women and followed the participants for 12 months after surgery at specific time points, *i.e.*, pre-surgery, 4–8 weeks post-surgery, and 12 months post-surgery. All the participants received general instructions (*The-Optimal-Lymph-Flow*^TM^: Keep a Healthy Weight) to strive for maintaining pre-surgery weight by encouraging nutrition-balanced (consumption of more vegetables and fruits) and portion-appropriate diet (cease eating when feeling 75% full for each meal) as well as daily or weekly physical activity [24]. Table 1 described the strategies, rationales, and actions to maintain pre-surgery weight.

### 2.3. Measures and Instruments

#### 2.3.1. Demographic and Medical Information

We gathered demographic and clinical information regarding breast cancer treatment, stage of disease, cancer location, type of adjuvant therapy, and treatment complications. Clinical information was verified by reviewing medical records.

#### 2.3.2. Height, BMI, and Body Compositions

Height was measured to the nearest 0.1 cm with a portable stadiometer (Scale-Tronix 5002 Stand on Scale, Scale-Tronix Company, Carol Stream, IL, USA) without shoes. An electrical bioimpedance device (InBody 520, Biospace Co., Ltd, Seoul, Korea) was used to measure weight, BMI, and percent of body fat. The device measures weight and automatically calculates BMI using the formula: weight (kg)/height (m^2^) and estimates the percent of body fat.

#### 2.3.3. Lymph Fluid Level

We used the Imp XCA® (Impedimed, Brisbane, Australia), a FDA approved Bioelectrical Impedance Analysis (BIA) device to assess lymph fluid level. The device measures impedance and resistance of the extracellular fluid in terms of L-Dex ratio. The L-Dex ratio ranges from −10 to 10, taking into consideration the ratio between dominant and non-dominant arms [23]. With the development of lymphedema, the impedance of the limb decreases and the L-Dex ratios increases. The L-Dex ratio ranges from −10 to 10, which is corresponding to the impedance ratio from 0.935 to 1.139 for at-risk dominant arms and 0.862 to 1.066 for at-risk non-dominant arms, respectively. Each one standard unit in L-Dex is equivalent to the impedance ratio of 0.03 [23].

### 2.4. Procedures

Researchers were trained to the procedure for obtaining informed consent and collecting data. After the institutional review board approved the study, patients were invited to participate in the study. The study invitation was distributed to potential patients by surgeons, oncologists, and nurses who cared for the patients. Written consent to the study was obtained from each participant. Procedures for using the bioimpedance devices were followed as recommended by the manufacturer and used in prior research [23,24].

### 2.5. Data Analysis

Data were analyzed using SPSS v22 (IBM Corp. Released 2013. IBM SPSS Statistics for Windows, Version 22.0. Armonk, NY, USA: IBM Corp.). Prior to analysis, data were examined for completeness and accuracy. Inaccuracies were reviewed by researchers and corrections made as necessary. Descriptive statistics were calculated for demographic and clinical characteristics including weight, weight change, BMI, and lymph fluid level by BIA.

**Table 1:** *The-Optimal-Lymph-Flow*^TM^: Keep a healthy weight.

Strategies	Rationales	Actions
➣ Eat nutrition-balanced diet (*i.e.*, more vegetables and fruits as well as quality proteins) ➣ Maintain portion-appropriate diet (feeling 75% full for each meal)	✓ Overweight or obesity is an important risk factor for lymphedema. ✓ Having extra weight makes it difficult for lymph flow and drain. This can lead to extra lymph fluid build-up.	✓ Each meal daily ✓ Although there are a lot of weight reduction programs, each person may respond differently to each program. ✓ It is important to talk to the nutritionist who can help to find a proper weight reduction programs.
➣ Stay hydrated	✓ People may actually be thirsty, not hungry	✓ Drink six to eight glasses of water daily; in the morning, before and during meals, and throughout the day. ✓ Avoid drinks with calories (e.g., juices). ✓ Drink green tea to boost metabolism.
➣ Large muscle exercises	✓ Daily large muscle exercises (e.g., walking, running, swimming, Yoga) help to burn more calories. ✓ Daily large muscle exercises also promote lymph flow by creating muscle pumps.	✓ At least 30-minutes three times a week or daily
➣ Get enough sleep	✓ Lack of sleep increases the production of the stress hormone cortisol, creates hunger, and leads to overeating. ✓ Getting just one more hour of sleep per night reduces belly fat accumulation.	✓ At least 7–8 hours of sleep per night.

Obesity was defined as BMI > 30 kg/m^2^, overweight as BMI 25.0–29.9 kg/m^2^, normal weight as BMI 18.5–24.9 kg/m^2^, and underweight <18.5 kg/m^2^ [1].Weight change was defined as a 5% gain or loss relative to pre-surgery weight, and was assessed at 4–8 weeks and 12 months post-surgery. Percent of weight change was calculated as: [pre-surgery weight (lb) − weight at 12-month post-surgery (lb)]/weight at 12-month post-surgery (lb) × 100. The changes in weight were then categorized into: no change (<5% weight loss or weight gain), weight gain (>5% increase), or weight loss (>5% decrease) [25]. Patients identified as underweight were categorized in the underweight/normal weight group due to the very small sample size of two patients.

In addition to treating L-Dex ratio as a continuous variable, we used the evidence-based cutoff point of >7.1 to determine the presence of lymphedema [23] since there are no existing data to support the sensitivity and specificity of BIA using L-Dex ratio >10 as the diagnostic cutoff point for lymphedema in clinical settings.

Means and standard deviations were used to summarize continuous variables, frequencies and percentages were used to summarize categorical variables. Clinical measures with repeated observations were plotted to examine trends over time for the entire sample and for subgroups. Pearson correlation coefficients were calculated to determine associations between study variables at each measurement time point. Welch’s robust ANOVA tests were conducted to compare average L-Dex ratio by weight status categories defined by BMI ranges. Games-Howell post-hoc tests were used for pairwise mean comparisons following any significant main effects.

## 3. Results

### 3.1. Participant Characteristics

The participants in the study were women with a mean age of 52 years (range: 26–81 years) with comparatively higher education, *i.e.*, 45.6% having a bachelor degree, 21.3% a graduate degree, and 33.1% an associate degree or high school diploma. More than one-half of the participants were married (58.8%) and the majority was employed (83.1%). The participants reported their race/ethnicity as white (60.3%), Asian (9.6%), black/African American (19.9%), or Hispanic (8.8%).

About one-half of the participants (48.5%) had lumpectomy, 40.4% had mastectomy with immediate reconstruction, only 11% had mastectomy alone. Among the participants who received chemotherapy, 70.7% had adjuvant chemotherapy after breast cancer surgery and 29.3% of the participants had neoadjuvant chemotherapy before cancer surgery. More than 70% of participants had radiation therapy and had an average of four lymph nodes removed (Table 2).

**Table 2:** Demographic and Clinical Characteristics by Pre-surgery Baseline BMI Category.

	Underweight/ Normal (≤24.9) (n = 50)		Overweight (25–29.9) (n = 44)		Obese (≥ 30) (n = 42)		*p*	Total (n = 136)	
**Age M(SD)**	48.9	10.9	51.3	10.9	56.8^†^	10.1	0.002*	52.1	11.1
	n	%	n	%	n	%	*p*	n	%
**Education**							0.085**		
Associates degree or less	12	24.0	34	34.7	5	29.4		45	33.1
Bachelor’s degree	27	54.0	22	50.0	13	31.0		62	45.6
Graduate degree	11	22.0	10	22.7	8	19.0		29	21.3
**Marital status**							0.377**		
Married/partnered	28	56.0	29	65.9	23	54.8		80	58.8
Divorced/Widowed	5	10.0	6	13.6	9	21.4		20	14.7
Single, never partnered	17	34.0	9	20.5	10	23.8		36	26.5
**Ethnicity**							0.137**		
Black/African American	7	14.0	6	13.6	14	33.3		27	19.9
White Non-Hispanic	31	62.0	30	68.2	21	50.0		82	60.3
Asian	8	16.0	2	4.5	3	7.1		13	9.6
Hispanic/Latino	4	8.0	5	11.4	3	7.1		12	8.8
Other	0	0	1	2.3	1	2.4		2	1.5
**Employment**							0.163**		
Unemployed	7	14.0	5	11.4	11	26.2		23	16.9
Employed	43	86.0	39	88.6	31	73.8		113	83.1
**Surgery**							0.166**		
Mastectomy	5	10.0	3	6.8	7	16.7		15	11
Lumpectomy	19	38.0	25	56.8	22	52.4		66	48.5
Mastectomy with immediate reconstruction	26	52.0	16	36.4	13	31.0		55	40.4
Nodes removed M(SD)	2.9	1.9	3.2	2.5	3.8	4.3		3.3	2.9
Nodes removed Median	2		2		3		0.053***	2	
**Chemotherapy (n = 75)**	(n = 28)		(n = 24)		(n = 23)		0.332**		
Neoadjuvant	10	35.7	8	33.3	4	17.4		22	29.3
Adjuvant	18	64.3	16	66.7	19	82.6		53	70.7
**Radiation**							0.232**		
No	18	36.7	13	31.0	7	19.4		38	29.9
Yes	31	63.3	29	69.0	29	80.6		89	70.1

^†^ Significantly different from underweight/normal weight group; * ANOVA; ** Fisher’s exact test; *** Kruskal-Wallis test.

### 3.2. Obesity and Weight Change

Average BMI was stable across measurement periods (pre-surgery BMI $\bar{x}$ = 27.7 kg/m^2^; range: 17.0–48.4), 4–8 weeks post-surgery BMI $\bar{x}$ = 27.6 kg/m^2^; range: 16.5 to 49.6), and 12 months post-surgery BMI $\bar{x}$ = 27.6 kg/m^2^; range: 17.4–48.4). There was no significant change in mean BMI or BMI categories over the 12-month period. Consistently, about 30% of participants (n = 42/136) were obese; more than 32% (n = 44/136) were overweight, and more than 37% (n = 50/136) were under- or normal weight. Only two participants had BMI < 18.5 and these two patients were included in the normal weight group for analyses (Table 3).

**Table 3:** Weight, BMI, and L-Dex ratios during 12-months.

	Pre		Post-Surgery (4–8 weeks)		Post-Surgery (12 months)	
	M	SD	M	SD	M	SD
Weight (lbs)	159.4	35.6	158.3	35.7	158.9	33.5
BMI	27.7	6.3	27.6	6.5	27.6	5.9
L-Dex Ratio	0.053	4.5	1.6	6.1	2.6	13.7

There were no significant differences in terms of demographic and clinical characteristics among participants in the normal/underweight, overweight, and obesity groups, except that participants in the obesity group were significantly older ($\bar{x}$ = 56.8 years of age) than those in the normal/underweight group ($\bar{x}$ = 48.9 years of age; *p* = 0.002).

Participants in the obesity group had a significantly higher percent of body fat in comparison with the overweight and normal/underweight groups. Consistently, participants in the obesity group had a mean percent of body fat of approximately 47% as compared to those in the overweight group who had a mean of 37% body fat, during the 12-month time period. Percent of body fat did not increase significantly during 12-month period post-surgery. There were significantly strong positive correlations between BMI and percent of body fat at each measurement point pre-surgery (r = 0.955; *p* < 0.01), 4–8 weeks post-surgery (r = 0.911; *p* < 0.01), and 12 months post-surgery (r = 0.891; *p* < 0.01).

The mean pre-surgery weight was 159.4 pounds (range: 99.0–266.3 pounds), 4–8 weeks post-surgery weight 158.3 pounds (range: 96.1–262.4 pounds), and 12-month post-surgery weight 158.9 pounds (range: 101.2–263.7 pounds). There was no significant change in average weight over 12-month period post-surgery (Table 3). At 4–8 weeks post-surgery assessment, the majority of participants (94.1%; n = 128/136) maintained their pre-surgery weight, while only 1.5% (n = 2/136) experienced an increase (>5%) weight and 4.4% (n = 6/136) experienced a loss (>5%). However, at 12 months post-surgery, 72.1% (n = 98/136) maintained their pre-surgery weight while 12.5% (n = 17/136) increased 5% of weight and 15% (n = 21/136) decreased 5% of their weight. Demographic and clinical characteristics were not associated with post-surgery weight change ≥ ±5%), except that more participants who gained >5% weight were more likely to have had neoadjuvant chemotherapy. At 12 months post-surgery, there were no significant differences in percent of body fat among weight change groups.

### 3.3. Lymph Fluid Level in Terms of L-Dex Ratio

There was a trend of increased lymph fluid level reflected in increasing L-Dex ratios during the 12-month period, yet this change was not significant. The mean pre-surgery L-Dex ratio was 0.05 (range: −9.8 to 12.9), 4–8 weeks post-surgery L-Dex ratio was 1.6 (range: −10.0 to 27.6), 12-month post-surgery L-Dex ratio was 2.6 (range: −10.6 to 96.9) (Table 3). The L-Dex ratio was significantly higher in the obesity group in comparison with the normal/underweight group at 4–8 weeks post-surgery, while there were no significant differences in the mean L-Dex ratio among the three groups at pre-surgery and 12 months post-surgery.

There were significant differences in the percentage of patients identified as having lymphedema based on an L-Dex >7.1 pre-surgery and 4–8 weeks post-surgery. A greater percentage of obese patients were identified as having lymphedema at each time point (Table 4). At pre-surgery, significantly higher occurrence of lymphedema defined by L-Dex ratios >7.1 (ranging 7.4 to 12.9) was observed in the obesity group (14.3%; n = 6/42). At 4–8 weeks post-surgery, significantly more participants in the obesity group had L-Dex ratios >7.1 (25.6%; n = 11/43). At 12-month post-surgery, there was a trend that more patients in the obesity group had L-Dex ratios >7.1 (19.5; n = 8/41) (Table 4).

**Table 4:** L-Dex ratios by BMI category.

	Underweight/ Normal weight (≤24.9)		Overweight (25–29.9)		Obese (≥30)		*p* Value
Pre-Surgery Baseline	(n = 50)		(n = 44)		(n = 42)		
	M	SD	M	SD	M	SD	*p*
L-Dex	−0.35	3.92	−0.37	4.53	0.97	5.04	0.335
	n	%	n	%	n	%	*p*^†^
L-Dex >7.1	0	0	2	4.5	6	14.3	0.009
							
Post-surgery (4–8 weeks)	(n = 53)		(n = 40)		(n = 43)		
	M	SD	M	SD	M	SD	*p*
L-Dex	−0.1	3.94	2.49	7.13	2.78*	6.91	0.018*
	n	%	n	%	n	%	*p*^†^
L-Dex >7.1	1	1.9	8	20.0	11	25.6	0.001
							
Post-surgery (12 months)	(n = 53)		(n = 42)		(n = 41)		
	M	SD	M	SD	M	SD	*p*
L-Dex	0.35	11.2	1.79	7.18	6.17	19.9	0.250
	n	%	n	%	n	%	*p*^†^
L-Dex >7.1	3	5.7	7	16.7	8	19.5	0.089

* Significantly different than Underweight/Normal weight; ^†^ Fisher’s exact.

Only two participants who had pre-surgery L-Dex ratios >7.1 sustained L-Dex ratios >7.1 at 4–8 weeks and 12 months post-surgery, while four participants improved their L-Dex ratios post-surgery. Two participants who had L-Dex ratios >20 at 4–8 weeks post-surgery had an increased L-Dex ratios of 78.6 and 96.9 at 12 months post-surgery, respectively. There were no significant correlations between L-Dex ratio and BMI, percent of body fat, and weight at pre-surgery, 4–8 weeks post-surgery, and 12 months post-surgery.

## 4. Discussion

In this prospective study, more than one in two women were obese or overweight at the time of pre-surgery, *i.e.*, more than 60% of the participants were obese (30.8%; n = 42/136) or overweight (32.4%; n = 44/136), while only two participants were underweight and about 35% (n = 50/136) of the participants had normal weight. This pattern of obesity and overweight was consistent at 4–8 weeks and 12 months post-surgery. There were significantly strong positive correlations between BMI and percent of body fat at pre-surgery (r = 0.955; *p* < 0.01), 4–8 weeks post-surgery (r = 0.911; *p* < 0.01); and 12 month post-surgery (r = 0.891; *p* < 0.01). Obesity is the accumulation of body fat, the strong positive correlations between BMI and percent of body fat indicate that BMI can be considered as an appropriate assessment for obesity. Given that being obese or overweight increases the risk of cancer recurrence, other chronic illnesses (such as type 2 diabetes, asthma, chronic back pain, osteoarthritis, and cardiovascular disease) [26,27] as well as lymphedema [18,20,21]; clearly obesity and overweight remain an important health concern for women who were diagnosed with, treated for, and survived breast cancer. Similar to prior research findings [25,26], our study also supports that special attention should be paid for the management of obesity and overweight in women who are older and those with less than university education.

Weight gain has been commonly reported by women treated for breast cancer [27,28,29,30]. Recent research shows that more than 60% of women treated for breast cancer had weight gain and more than 47% had at least 5% weight gain [25]. Currently, it is unknown whether post-diagnosis weight loss can lead to beneficial improvement for prognosis and disease- free survival. Maintaining pre-surgery weight is key to preventing adverse treatment related effects and other related co-morbid conditions for women treated for breast cancer [24,25]. It is premature to advocate for weight loss programs for women at the time of breast cancer diagnosis. Perhaps, the appropriate recommendation is to provide women with general instructions, as we did in our study, on having nutrition-balanced and portion-appropriate diet and physical activity daily or weekly. Such general instructions were effective for our patients. In our study, 72.1% maintained their pre-surgery weight and 15.4% of women had >5% weight loss while only 12.5% had an increase >5% of their weight at 12 months post-surgery.

Although the exact pathophysiological basis of weight gain following breast cancer remains unclear, chemotherapy has been reported as a significant contributing factor for weight gain after breast cancer diagnosis [31]. Findings of our study show that there were no significant differences in any demographic or clinical characteristic among women who maintained pre-surgery weight, lost >5% weight, or gained >5% weight, except that significantly more women who received neoadjuvant chemotherapy prior to surgery had >5% weight increase at 12 months post-surgery. More prospective population-based cohort studies are needed to better understand the likely contributing factors to weight gain following breast cancer treatment.

Research has provided the evidence regarding the association between BMI > 30 kg/m^2^ and lymphedema defined by perometer measure of >10% limb volume increase in comparison with pre-surgery baseline measurement of limb volume [13,14,15,16,17,18,19,20,21,22]. The best evidence in the existing studies is the use of indirect measure of lymphedema in terms of limb volume by perometer [18,21]. To the best of our knowledge our study was the first to prospectively examine BMI and lymph fluid level using BIA. In this way, the use of BIA at meaningful time points, *i.e.*, pre-surgery, 4–8 weeks and 12 months post-surgery, provides strength to our study.

It should be noted that obesity defined by >30 BMI kg/m^2^ is related to lymphedema defined by >7.1 L-Dex ratio by BIA [23]. Significantly more patients in the obesity group had L-Dex ratios >7.1 than those in the normal/underweight and overweight group at pre-surgery, and 4–8 weeks post-surgery. Further, there was a trend of more patients in the obesity group for L-Dex ratios >7.1 as compared to those in the normal/underweight and overweight group at 12-months post-surgery. In fact, two patients who developed severe lymphedema with L-Dex ratios of 78.6 and 96.9 at 12 months post-surgery also had BMI > 30 kg/m^2^. It is interesting that more patients in the obesity group had L-Dex ratios >7.1 at pre-surgery. However, four out of six patients improved their L-Dex ratios at 4–8 weeks and 12 months post-surgery. The pathophysiology underlying the increased L-Dex ratio for obese patients needs to be further explored.

It should be noted that significantly higher L-Dex ratios were observed in the obesity group with BMI > 30 kg/m^2^ at 4–8 weeks post-surgery. The two patients, who developed severe lymphedema at 12-months post-surgery had pre-surgery L-Dex ratios of 0.5 and 2.0, which increased to L-Dex ratios of 22.7 and 20.2 at 4–8 weeks post-surgery, eventually had L-Dex values of 96.9 and 78.6 at 12 months post-surgery, respectively. While large population-based research is needed to ascertain whether L-Dex values at pre-surgery and 4–8 weeks post-surgery are predictive of late severe lymphedema, perhaps, attention should be paid to patients who had L-Dex ratios >20 at 4–8 weeks post-surgery. No significant correlations between L-Dex value and BMI, percent of body fat, and weight, were found at any time point. This may be due to a combination of the small sample size and high degree of variability in individual measures. Population-based and prospective research is needed to further discern the relationship between BMI and lymph fluid level measured by BIA.

## 5. Conclusions

Obesity and overweight are health risks among a substantial proportion of women at the time of cancer diagnosis and the patterns of obesity and overweight continue during the first year of treatment. While it is unknown whether post-diagnosis weight loss is beneficial to disease-free survival and prevention of comorbidities, it is appropriate to encourage women to make every effort to maintain pre-surgery weight to prevent occurrence of adverse treatment-related effects such as lymphedema, since obesity does have influence on lymph fluid level and limb volume. General instructions on having a nutrition-balanced and portion-appropriate diet and physical activity daily or weekly can be effective to maintain pre-surgery weight. Such general instructions may create less burden and stress to women when facing the diagnosis and treatment of breast cancer.

## Abbreviations

BMI: body mass index
L-Dex: lymphedema index
BIA: bioelectrical impedance analysis

## References

[1] World Health Organization Obesity and overweight fact sheet N°311 updated August 2014. http://www.who.int/mediacentre/factsheets/fs311/en/.

[2] Jemal M., Bray F., Center M.M., Ferlay J., Ward E., Forman D. (2011). Global cancer statistics. Cancer J..

[3] Chan D.M., Vieria A.R., Aune D., Bandera E.V., Greenwood D.C., McTiernan A., Navarro Rosenblatt D., Thune I., Vieira R., Norat T. (2014). Body mass index and survival in women with breast cancer—Systematic review and meta-analysis of 82 follow-up studies. Ann. Oncol..

[4] Chen X., Lu W., Gu K., Chen Z., Zheng Y., Zheng W., Shu X.O. (2011). Weight change and its correlates among breast cancer survivors. Nutr. Cancer.

[5] Chen X., Lu W., Zheng W., Gu K., Chen Z., Zheng Y., Shu X.O. (2010). Obesity and weight change in relation to breast cancer survival. Breast Cancer Res. Treat..

[6] American Cancer Society (2013). Breast Cancer Facts and Figures 2013–2014.

[7] Fu M.R., Deng J., Armer J. (2014). Cancer-related lymphedema: Evolving evidence for treatment and management from 2009 to 2014. Clin. J. Oncol. Nurs..

[8] McLaughlin A., Wright M.J., Morris K.T., Giron G.L., Sampson M.R., Brockway J.P., Hurley K.E., Riedel E.R., van Zee K.J. (2008). Prevalence of lymphedema in women with breast cancer 5 years after sentinel lymph node biopsy or axillary dissection: Objective measurements. J. Clin. Oncol..

[9] Paskett E.D., Naughton M.J., McCoy T.P., Case L.D., Abbott J.M. (2007). The epidemiology of arm and hand swelling in premenopausal breast cancer survivors. Cancer Epidemiol. Biomarkers Prev..

[10] Petrek J.A., Senie R.T., Peters M., Rosen P.P. (2001). Lymphedema in a cohort of breast carcinoma survivors 20 years after diagnosis. Cancer.

[11] Fu R.M. (2014). Breast cancer-related lymphedema: Symptoms, diagnosis, risk reduction, and management. J. Clin. Oncol..

[12] Disipio T., Rye S., Newman B., Hayes S. (2013). Incidence of unilateral arm lymphoedema after breast cancer: A systematic review and meta-analysis. Lancet Oncol..

[13] Tsai R.J., Dennis L.K., Lynch C.F., Snetselaar L.G., Zamba G.K., Scott-Conner C. (2009). The risk of developing arm lymphedema among breast cancer survivors: A meta-analysis of treatment factors. Ann. Surg. Oncol..

[14] Norman S.A., Localio A.R., Kallan M.J., Weber A.L., Torpey H.A., Potashnik S.L., Miller L.T., Fox K.R., DeMichele A., Solin L.J. (2010). Risk factors for lymphedema after breast cancer treatment. Cancer Epidemiol. Biomarkers Prev..

[15] Graham P., Jagavkar R., Browne L., Millar E. (2006). Supraclavicular radiotherapy must be limited laterally by the coracoid to avoid significant adjuvant breast nodal radiotherapy lymphoedema risk. Australas. Radiol..

[16] Shih Y.C., Xu Y., Cormier J.N., Giordano S., Ridner S.H., Buchholz T.A., Perkins G.H., Elting L.S. (2009). Incidence, treatment costs, and complications of lymphedema after breast cancer among women of working age: A 2-year follow-up study. J. Clin. Oncol..

[17] Cormier J.N., Xing Y., Zaniletti I., Askew R.L., Stewart B.R., Armer J.M. (2009). Minimal limb volume change has a significant impact on breast cancer survivors. Lymphology.

[18] Ridner S.H., Dietrich M.S., Stewart B.R., Armer J.M. (2011). Body mass index and breast cancer treatment-related lymphedema. Support. Care Cancer.

[19] Kwan M.L., Darbinian J., Schmitz K.H., Citron R., Partee P., Kutner S.E., Kushi L.H. (2010). Risk factors for lymphedema in a prospective breast cancer survivorship study: The pathways study. Arch. Surg..

[20] Helyer L.K., Varnic M., Le L.W., Leong W., McCready D. (2010). Obesity is a risk factor for developing postoperative lymphedema in breast cancer patients. Breast J..

[21] Jammallo L.S., Miller C.L., Singer M., Horick N.K., Skolny M.N., Specht M.C., O’Toole J., Taghian A.G. (2013). Impact of body mass index and weight fluctuation on lymphedema risk in patients treated for breast cancer. Breast Cancer Res. Treat..

[22] Goldberg J.I., Wiechmann L.I., Riedel E.R., Morrow M., van Zee K.J. (2010). Morbidity of sentinel node biopsy in breast cancer: The relationship between the number of excised lymph nodes and lymphedema. Ann. Surg. Oncol..

[23] Fu M.R., Cleland C.M., Guth A.A., Kayal M., Haber J., Cartwright F., Kleinman R., Kang Y., Scagliola J., Axelrod D. (2013). L-Dex ratio in detecting breast cancer-related lymphedema: Reliability, sensitivity, and specificity. Lymphology.

[24] Fu M.R., Axelrod D., Guth A., Cartwright F., Qiu Z., Goldberg J., Kim J., Scagliola J., Kleinman R., Haber J. (2014). Proactive approach to lymphedema risk reduction: A prospective study. Ann. Surg. Oncol..

[25] Yaw Y.H., Kandiah M., Shariff Z.M., Mun C.Y., Hashim Z., Yusof R.M., Othman Z., Saibul N., Weay Y.H. (2010). Pattern of weight changes in women with breast cancer. Asian Pac. J. Cancer Prev..

[26] Demark-Wahnefried W., Campbell K., Hayes S.C. (2012). Weight management and its role in breast cancer rehabilitation. Cancer.

[27] Jiralerspong S., Kim E.S., Dong W., Feng L., Hortobagyi G.N., Giordano S.H. (2013). Obesity, diabetes, and survival outcomes in a large cohort of early-stage breast cancer patients. Ann. Oncol..

[28] Makari-Judson G., Braun B., Jerry D.J., Mertens W.C. (2014). Weight gain following breast cancer diagnosis: Implication and proposed mechanisms. World J. Clin. Oncol..

[29] Gu K., Chen X., Zheng Y., Chen Z., Zheng W., Lu W., Shu X.O. (2013). Weight change patterns among breast cancer survivors: Results from the Shanghai breast cancer survival study. Cancer Causes Control.

[30] Irwin M.L., McTiernan A., Baumhartner R.N., Baumgartner K.B., Bernstein L., Gilliland F.D., Ballard-Barbash R. (2005). Changes in body fat and weight after a breast cancer diagnosis: Influence of demographic, prognostic, and lifestyle factors. J. Clin. Oncol..

[31] Wang J.S., Cai H., Wang C.Y., Zhang J., Zhang M.X. (2014). Body weight changes in breast cancer patients following adjuvant chemotherapy and contributing factors. Mol. Clin. Oncol..

